# Platelet RNA signatures for the detection of cancer

**DOI:** 10.1007/s10555-017-9674-0

**Published:** 2017-07-05

**Authors:** Nik Sol, Thomas Wurdinger

**Affiliations:** 1 Department of Neurology, VU University Medical Center, Amsterdam, The Netherlands; 2 Brain Tumor Center Amsterdam, VU University Medical Center, Amsterdam, The Netherlands; 3 Department of Neurosurgery, VU University Medical Center, Amsterdam, The Netherlands; 4 Department of Neurology, Massachusetts General Hospital and Neuroscience Program, Harvard Medical School, Boston, MA USA

**Keywords:** Platelets, Transcriptome, mRNA, Splicing, Tumor-educated platelets, Liquid biopsy, Biomarkers

## Abstract

Platelets are equipped with RNA processing machineries, such as pre-mRNA splicing, pre-miRNA processing, and mRNA translation. Since platelets are devoid of a nucleus, most RNA transcripts in platelets are derived from megakaryocytes during thrombocytogenesis. However, platelets can also ingest RNA molecules during circulation and/or interaction with other cell types. Since platelets were first described by Bizzozero in 1881, their well-established role in hemostasis and thrombosis has been intensively studied. However, in the past decades, the list of biological processes in which platelets play an important role keeps expanding. In this review, we discuss how platelet RNA biomarker signatures can be altered in the presence of cancer.

## Platelets and cancer in brief

**Fig. 1:**
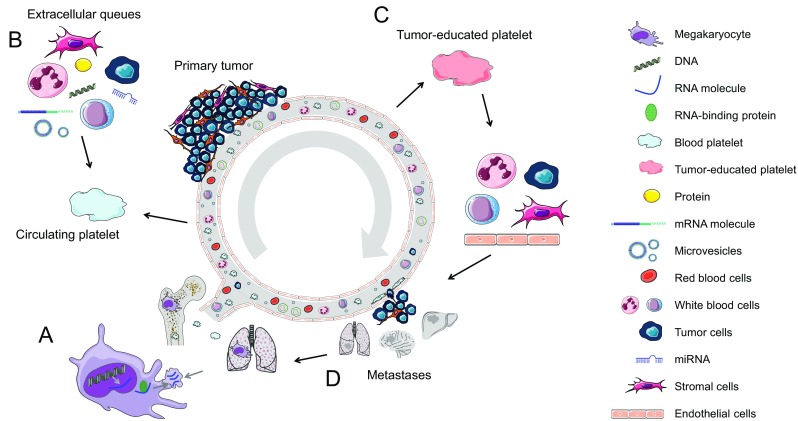
Schematic representation of tumor-mediated education of platelets and the megakaryocyte leading to metastasis. (**a**) Megakaryocytes in the bone marrow and lungs sort specific RNA and proteins into platelet precursors. (**b**) Circulating platelets contain a variety of RNA transcripts and proteins. During their 7–10-day lifespan, platelets interact with immune cells, cancer cells, and stromal cells. These direct interactions as well as distant cell signaling, for instance, *via* vesicle-mediated communication in whole blood, changes the content of the platelet and platelet function. (**c**) This process leads to the development of tumor-educated platelets. Next, tumor-educated platelets can influence the process of metastasis formation by stimulating or blocking immune cells, endothelial cells, stromal cells, and cancer cells, either by direct cell-to-cell contact or by releasing extracellular queues. (**d**) Finally, metastasis could affect the sorting of specific RNA and proteins of megakaryocytes into platelets

Platelets are implicated in tumor biology and metastasis (Fig. 1) [1, 2], and tumor cells can, directly and indirectly, impose changes on platelet RNA and protein content [3–5]. As a result, these tumor-educated platelets (TEPs) have an altered function and can in various ways promote tumor cell survival and metastasis, as well as other hallmarks of cancer [6]. Tumor cells interact with platelets indirectly *via* different signaling molecules or directly *via* different receptors, mainly the platelet activation receptor P-selectin [7–10]. Upon activation, platelets can release several growth and pro-angiogenic factors like ANGPT1, PDGF, BFGF, EGF, HGF, IGF1, TGFβ, VEGF-A, and VEGF-C [11, 12]. Platelets can release these factors at a metastatic niche, thereby providing a pro-tumoral growth microenvironment [1].

Calverley et al. showed that NAD-dependent deacetylase sirtuin-2 (SIRT2) is differentially spliced in platelets of metastatic lung cancer patients. This gene plays a role in epigenetic silencing, concluding that platelets could induce growth and progression of tumors by releasing epigenetic silencers [5]. Further, platelets alter cancer cells to evade the detection by the immune system, by transferring MHC class I proteins to the tumor cells, resulting in protection against natural killer cells [13]. Platelets can also mechanically protect cancer cells from destruction *via* NK cells by forming a cell–fibrin–platelet aggregate surrounding circulating tumor cells (CTC) or arrested tumor cells. This physical shield avoids cell-mediated immune detection and supports cancer cell survival [14–17]. Such protective properties of platelets are important for cancer metastasis by promoting cancer cell survival in the circulation [18, 19].

## Liquid biopsies and cancer detection

Liquid biopsies have been introduced as a potential game changer in cancer management, with blood tests emerging as a minimally invasive, safe, and sensitive alternative or complementary approach for tissue biopsies [20–22]. Blood represents a rich source of information through which solid cancers (and their subtypes) can be detected, identified and classified, and matched to a specific therapy [23–30]. Targeted risk-based screening based on a person’s individual risk of cancer is envisioned to be the anti-cancer strategy of the future. Current clinical oncology practice relies on the removal of tumor tissue through biopsies for analysis of tumor-linked genetic alterations. Although the use of tumor tissue biopsies is the current gold standard for cancer diagnosis and represents an essential tool in cancer management, it has become apparent that the information acquired from a single biopsy provides a spatially and temporally limited snapshot of a (metastatic) tumor and often fails to reflect the heterogeneity of the disease [22, 31, 32]. Moreover, tumor biopsies are invasive which poses a limitation for repeated sampling (needed for monitoring treatment response and resistance to targeted therapies). Liquid biopsies could provide a potential revolution in cancer diagnostics as a minimally invasive method for detecting and monitoring diseases, complementary to current tissue biopsy approaches. Liquid biopsies can therefore provide an accurate and comprehensive spatiotemporal snapshot of the tumor and its microenvironment on multiple levels, and enable (1) early detection (screening), (2) prognosis for the individual patient: stage and spread, (3) identification of new targets for personalized treatment, (4) pre-treatment classification for personalized therapy/prediction of response to therapy, (5) early therapy response monitoring, “real-time” assessment of treatment effectiveness, and (6) follow-up and early detection of recurrence of the disease and its metastases. Currently, blood-based biopsy measurements focus on evaluation of biomarker biosources, including circulating tumor DNA (ctDNA), circulating tumor cells (CTCs), extracellular vesicles (EVs; exosomes, microvesicles, microparticles, oncosomes), and tumor-educated platelets (TEPs) [3, 4, 33–38].

## Tumor-educated platelets

Platelets have long been considered as a potential diagnostic tool in cancer. Several studies have shown that a simple platelet count already harbors potential clinical relevant information [39–44]. Besides platelet counts, the size of platelets [44, 45] and platelet protein markers, such as P-selectin, are used for blood-based cancer diagnostics and prognostics [44, 46–51]. Platelets can interact with cancer cells in various ways leading to platelet hyper-reactivity [52, 53]. Furthermore, there may be an increase in young reticulated platelets in cancer patients [54]. The ratio of these young platelets in the total platelet population can change again after cancer treatment [43, 48, 55]. These observations indicate that platelets can respond reactively during tumor progression and treatment.

Tumor-associated biomolecules are transferred to platelets resulting in their “education” [3, 4, 56]. External stimuli, such as activation of platelet surface receptors and lipopolysaccharide-mediated platelet activation, induce specific splicing of pre-messenger RNAs (mRNAs) in circulating TEPs [57]. TEPs may also undergo queue-specific splice events in response to signals released by cancer cells and the tumor microenvironment such as by stromal and immune cells [1]. The combination of specific splice events in response to external signals and the capacity of platelets to directly ingest (spliced) circulating mRNA can provide TEPs with a highly dynamic mRNA repertoire, with potential applicability to cancer diagnostics. A highly sensitive method for isolation and analysis of TEP RNA was developed to detect cancer. It was shown that platelet mRNA profiles can be used to distinguish between healthy donors and cancer patients [3–5]. Given the many roles platelets play in cancer, they could harvest information about the disease status of given patients.

Platelets are capable of taking up protein and nucleotides during their lifespan. Nilsson et al. described the uptake of extracellular vesicles (EVs) by circulating platelets [3]. *Via* this mechanism, platelets can sequester EVs from cancer cells harboring tumor-specific RNA. EGFRvIII, a deletion mutant of the epidermal growth factor receptor (EGFR), is such a specific tumor RNA which is considered to be present in 30% of glioblastoma tumors. Traces of this very malignant tumor of the central nervous system could be detected by RT-PCR of platelets from these patients. The EGFRvIII RNA transcript was detected with a sensitivity of 80% (four out of five EGFRvIII-positive tumors were detected), and a specificity of 96% (25 out of 26 EGFRvIII-negative tumors were scored as negative). In addition, microarray analysis discovered an RNA signature that could distinguish between glioblastoma patients (*n* = 8) and healthy controls (*n* = 12) [3]. A total of 17 out of the top 30 differentially expressed genes were also found in the mRNA sequencing data from TEPs of GBM patients, with four of the most significantly differentially expressed, WFDC1, FKBP5, IL1R2, and TPCN1. In 2015, the uptake of tumor-derived RNA in platelets was confirmed in NSCLC patients. Translocated EML4-ALK transcripts and KRAS and EGFR transcripts harboring tumor-specific point mutations were detected by RT-PCR and deep amplicon sequencing [4, 33]. Although shallow sequencing of platelets did not reveal the specific mutations, the RNA profiles allowed for the development of surrogate gene panels to determine the molecular status of the tumor tissue *in situ*.

## TEP RNA signatures

Microarray mRNA platelet profiles of seven healthy donors and five treatment-naive metastatic lung cancer patients were compared by Calverley et al. [5]. Unsupervised hierarchical clustering revealed 200 altered RNAs, of which all but 3 were decreased in platelets of lung cancer patients. They also showed that NAD-dependent deacetylase sirtuin-2 (SIRT2) is differentially spliced in platelets of metastatic lung cancer patients compared to healthy individuals. SIRT2 is a human homolog to yeast Sir2 protein that regulates epigenetic gene silencing and suppresses rDNA recombination and is a cellular stress response. In yeast, it regulates the acetylation status of several tumor suppressors including p53 and FoxO1; however, in humans its exact function is not yet known.

**Fig. 2:**
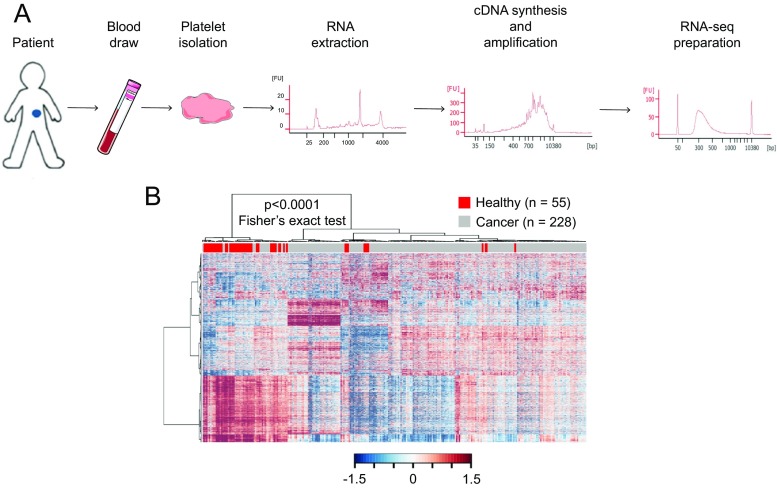
ThromboSeq workflow. (**a**) TEP mRNA sequencing workflow; blood draw is performed on patients, and from a single 6-ml EDTA-coated tube, platelets are isolated. RNA extraction is done according to manufacturer’s protocol using the mirVana RNA isolation kit (Life Technologies). Next, mRNA is amplified using SMARTer Ultra low input RNA kit (Clontech). Samples are prepared for sequencing on the Hiseq 2500 Illumina platform using the Truseq Nano DNA Sample Prep Kit (Illumina). After each step, quality control was performed by critical inspection of Bioanalyzer profiles (Agilent). (**b**) Heatmap of unsupervised clustering of platelet mRNA profiles of healthy donors (*red*, *n* = 55) and patients with cancer (*gray*, *n* = 228)

**Fig. 3:**
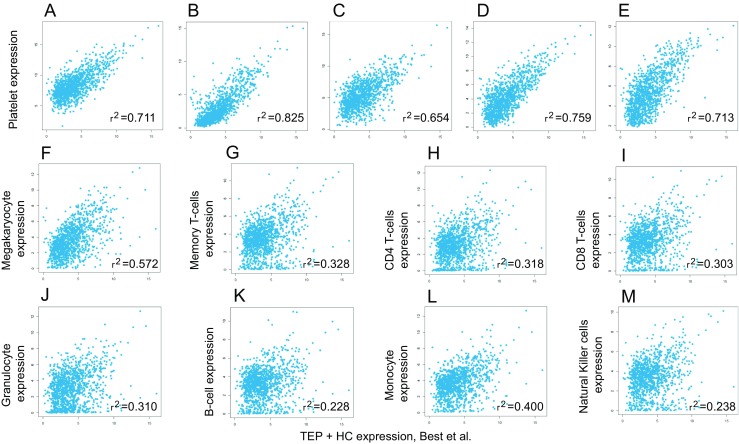
Correlation plots of TEP RNA signatures with other (**a**) nucleated blood cells. Correlation plots between platelets sequenced by Best et al. compared with platelet RNA expression levels from different studies and compared with RNA expression from different blood cells. (**a**) Platelets from Bray et al. [82]. (**b**) Poly A selected RNAs from platelets from Kissopoulou et al. [83]. (**c**) Ribosomal RNA-depleted platelets from Kissopoulou et al. [83]. (**d**) Platelets from Rowley et al. [84]. (**e**) Platelets from Simon et al. [85]. (**f**) Megakaryocyte [86]. (**g**) Memory T-cells [87]. (**h**) CD4 T-cells [87]. (**i**) CD8 T-cells [87]. (**j**) Granulocytes [87]. (**k**) B-cells [87]. (**l**) Monocytes [87]. (**m**) Natural killer cells [87]

A recent study demonstrated that mRNA sequencing of tumor-educated platelets distinguishes cancer patients from healthy individuals with 96% accuracy (Fig. 2) [4]. Blood contains 200–500 million platelets per milliliter, making platelets highly available for diagnostic use. Whole blood can be stored up to 48 h at room temperature prior to platelet isolation while maintaining high-quality RNA and the dominant cancer RNA signatures. Platelet isolation was performed with a two-step centrifugation protocol, and platelet purity was confirmed by manual cell counting using crystal violet staining on a light microscope showing one to five nucleated cells per 10 million platelets. Nucleated cells are considered to contain approximately 10,000× more RNA than a single platelet. However, nucleated cell counts of one to five nucleated cells per 10 million platelets indicate that the RNA profiles that were observed in the platelet fraction are likely not attributable to the presence of nucleated cell contamination. Furthermore, *in silico* analysis showed a high correlation between TEP RNA profiles with previously described platelet RNA profiles, which were derived from platelet isolations using a CD45 depletion step to further diminish possible leukocyte contamination, and no correlation with nucleated blood cells (Fig. 3). In addition, based on DAVID Gene Ontology analysis, the detected RNAs are strongly enriched for transcripts associated with blood platelets. Among the 5003 RNAs, known platelet markers were identified, such as B2M, PPBP, TMSB4X, and PF4 in high levels [4].

RNA sequencing was performed on 283 blood platelet samples, isolated from healthy individuals (*n* = 55) and patients with cancer (*n* = 228, cancer types: glioblastoma, non-small cell lung cancer, colorectal cancer, pancreatic cancer, breast cancer, and liver and bile duct carcinoma) [4]. A total of 1453 out of 5003 mRNAs were increased and 793 out of 5003 mRNAs were decreased in TEPs as compared to platelet samples of healthy donors while presenting a strong correlation between these platelet mRNA profiles. Unsupervised hierarchical clustering based on the differentially detected platelet mRNAs distinguished two sample groups with minor overlap. Using the different mRNA profiles of cancer patients and healthy donors, it was possible to develop a predictive algorithm with high accuracy in separating healthy individuals from cancer patients. The RNA profiles allowed for support vector machine (SVM) classification, enabling the ability to correctly classify whether the patient has cancer or not (accuracy: 96%), which tumor type is present (accuracy 71%), and which molecular mutational subtype the tumor has (accuracy 85–95%). Interestingly, all 39 patients with early-stage, non-metastasized cancer were correctly identified as cancer patients [4]. These promising data show that the TEP cancer classification platform deserves thorough follow-up evaluation experiments, profiling of additional prospectively collected patient cohorts, cancer (molecular) subtypes, and other (inflammatory) diseases, and further training and development of the cumulative SVM algorithms. This patient cohort included six (heterogenic) tumor types, i.e., non-small cell lung carcinoma (*n* = 60), colorectal cancer (*n* = 41), glioblastoma (*n* = 39), pancreatic cancer (*n* = 35), hepatobiliary cancer (*n* = 14), and breast cancer (*n* = 39).

Although it was not possible to measure significant differences between localized and metastasized tumors, it is not excluded that a larger sample set of localized and metastasized samples from the same cancer type will have the power to do so. Since the TEP profiles closely resemble the different tumor types as determined by their organ of origin, regardless of systemic dissemination, it was possible to develop a multiclass algorithm predicting the primary tumor location and separate tumors based upon their mutational status [4]. Different primary tumors showed different RNA profiles, making it possible to determine malignant lesions as a primary tumor or as a metastasis, for instance on chest imaging. Resemblance in platelet RNA profiles from different primary tumors is partly explained by similar driver mutations. KRAS for instance is a driver mutation in many cancer types. This mutation and others leave a specific signature in platelets making it possible to select patients for different targeted therapies. Furthermore, when sequenced deep enough, the specific driver mutations can be found in platelets.

This thromboSeq technique shows the potential of platelets as liquid biopsy biosource. Besides mRNA, platelets also contain non-coding and small RNAs. Analysis of differentially expressed non-coding RNAs revealed 20 genes of which 16 were upregulated in TEPs (Table 1). These 20 non-coding RNAs showed a tumor-specific profile. Interestingly, one of the downregulated RNAs is Metastasis Associated Lung Adenocarcinoma Transcript 1 (MALAT1) [4]. This non-coding RNA is retained in the nucleus where it acts as a transcriptional regulator of numerous genes, including some genes involved in cancer metastasis. Its upregulation in multiple cancerous tissues has been associated with the proliferation and metastasis of tumor cells [58–60]. Growth arrest-specific transcript 5 (GAS5), another downregulated non-coding RNA, is involved in cellular proliferation, and its downregulation has been shown to be pro-cancerous in several tumor types [61, 62]. Both SNHG5 (small nucleolar RNA host gene) and SNHG8 appear to play a role in gastric cancer by regulating migration and proliferation [63–65]. Lymphocytic leukemia 1 and 2 (DLEU1 and DLEU2) are frequently deleted in several hematological cancers, and cancer susceptibility candidate 15 (CASC15) has a role in the formation of neuroblastoma [66–68]. Given the function that these non-coding RNAs seem to have in cancer, it is interesting to further investigate non-coding RNA expression in platelets from cancer patients.

**Table 1:** Differentially expressed non-coding RNAs between healthy donor platelets and TEPs

Ensembl gene ID	HGNC symbol	Description	Chromosome	Band	Strand	Start position	End position	Coding gene size	logFC	logCPM	PValue	FDR
ENSG00000251562	MALAT1	Metastasis-associated lung adenocarcinoma transcript 1 (non-protein coding) [Source: HGNC Symbol; Acc:29665]	11	q13.1	1	65265233	65273940	8708	1.9466091726	3.3594222629	2.22E−18	1.36E−16
ENSG00000234741	GAS5	Growth arrest-specific 5 (non-protein coding) [Source: HGNC Symbol; Acc:16355]	1	q25.1	−1	173833038	173838020	3631	1.2965700312	6.0667784925	6.46E−18	3.75E−16
ENSG00000253394	LINC00534	Long intergenic non-protein coding RNA 534 [Source: HGNC Symbol; Acc:43643]	8	q21.3	1	91233716	91581546	1354	−1.2741948742	6.9347392686	6.66E−18	3.84E−16
ENSG00000233093	LINC00892	Long intergenic non-protein coding RNA 892 [Source: HGNC Symbol; Acc:48578]	X	q26.3	1	135721702	135724588	1918	−0.9799176457	5.6522502356	9.05E − 15	3.17E−13
ENSG00000237803	LINC00211	Long intergenic non-protein coding RNA 211 [Source: HGNC Symbol; Acc:37459]	2	p22.2	−1	38053390	38103417	2595	−0.8369418324	6.0623819217	3.19E−14	1.02E−12
ENSG00000224805	LINC00853	Long intergenic non-protein coding RNA 853 [Source: HGNC Symbol; Acc:43716]	1	p33	1	47644922	47646011	661	−0.8077160792	5.7337580102	1.59E−13	4.57E−12
ENSG00000269893	SNHG8	Small nucleolar RNA host gene 8 (non-protein coding) [Source: HGNC Symbol; Acc:33098]	4	q26	1	119199864	119200978	923	1.3323081879	3.4509009322	9.35E−12	2.03E−10
ENSG00000222041	LINC00152	Long intergenic non-protein coding RNA 152 [Source: HGNC Symbol; Acc:28717]	2	p11.2	1	87754887	87906324	3125	−0.7360299076	7.5625603844	2.60E − 11	5.10E−10
ENSG00000203875	SNHG5	Small nucleolar RNA host gene 5 (non-protein coding) [Source: HGNC Symbol; Acc:21026]	6	q14.3	−1	86370710	86388451	2206	1.1865024192	6.4001568217	5.55E−11	1.03E−09
ENSG00000174365	SNHG11	Small nucleolar RNA host gene 11 (non-protein coding) [Source: HGNC Symbol; Acc:25046]	20	q11.23	1	37075221	37079564	2813	−1.2708676941	4.2727590978	9.28E−11	1.65E−09
ENSG00000176124	DLEU1	Deleted in lymphocytic leukemia 1 (non-protein coding) [Source: HGNC Symbol; Acc:13747]	13	q14.2	1	50656307	51297372	9926	−0.8000576828	4.3696475017	8.62E − 10	1.29E−08
ENSG00000253819	LINC01151	Long intergenic non-protein coding RNA 1151 [Source: HGNC Symbol; Acc:49471]	8	q24.13	−1	123682624	123706345	571	−0.7125873249	5.4683635079	4.38E−09	5.72E−08
ENSG00000172965	MIR4435–1HG	mIR4435-1 host gene (non-protein coding) [Source: HGNC Symbol; Acc:35163]	2	q13	−1	111953927	112252677	8500	−0.5543385259	8.5284464047	2.25E−08	2.55E−07
ENSG00000232065	LINC01063	Long intergenic non-protein coding RNA 1063 [Source: HGNC Symbol; Acc:49092]	3	q29	−1	196358369	196359458	348	−0.8960016404	3.6110058313	3.23E−08	3.55E−07
ENSG00000215483	LINC00598	Long intergenic non-protein coding RNA 598 [Source: HGNC Symbol; Acc:42770]	13	q14.11	−1	41025131	41055143	1037	−0.8601585239	3.7626854672	1.02E−06	8.05E−06
ENSG00000237854	LINC00674	Long intergenic non-protein coding RNA 674 [Source: HGNC Symbol; Acc:44355]	17	q24.2	1	66098049	66111659	734	−0.7884907133	5.1088601129	2.63E−06	1.89E−05
ENSG00000250334	LINC00989	Long intergenic non-protein coding RNA 989 [Source: HGNC Symbol; Acc:48918]	4	q21.21	1	80413570	80497614	2231	−0.4460106539	9.2584443266	5.38E−06	3.62E−05
ENSG00000214194	LINC00998	Long intergenic non-protein coding RNA 998 [Source: HGNC Symbol; Acc:48953]	7	q31.1	−1	112756773	112758668	1896	−0.5153540079	4.2695677376	7.12E−05	3.57E−04
ENSG00000272168	CASC15	Cancer susceptibility candidate 15 (non-protein coding) [Source: HGNC Symbol; Acc:28245]	6	p22.3	1	21665003	22214734	11,161	−0.5758874127	5.1721135489	1.63E-04	7.36E−04
ENSG00000231607	DLEU2	Deleted in lymphocytic leukemia 2 (non-protein coding) [Source: HGNC Symbol; Acc:13748]	13	q14.2	−1	50601269	50699856	2728	−0.4586998033	6.4034037922	1.02E-04	4.91E−04

Of the non-coding RNAs, microRNAs are of special interest. Ple et al. described in 2012, 532 different micro-RNAs in platelets [69]. Increasing work suggests an important role for platelet microRNAs in platelet biogenesis and function [70–73]. In cardiovascular disease, nine differentially expressed microRNAs were found comparing patients with myocardial infarction with healthy controls [74]. This shows the potential of microRNAs in platelets as a diagnostic tool. Although microRNAs play an important role in cancer, their expression is mostly studied in tissue and exosomes. MicroRNA expression in TEPs needs further research to determine their diagnostic power.

DAVID and CAGE gene ontology algorithms were applied to the diagnostic RNA panels of tumor-educated platelets. This revealed a downregulation of RNAs involved in RNA metabolism and RNA splicing. Interestingly, there was a correlation to platelet activation, platelet and vesicle transport, cytoskeleton activation, and ATP signaling. These programs potentially reveal a hyperactive state of tumor-educated platelets matching the hyper-reactivity found in a functional analysis of platelets from certain cancer patients. This hyperactivity seems to be different in cancer patients compared to patients with non-cancerous inflammatory diseases. Microarray studies performed on platelets from different non-cancerous inflammatory diseases identified 22 differentially expressed genes [75–78]. Expression levels of these genes in TEPs appeared to be randomly expressed compared to platelets of healthy donors, suggesting that platelet RNA in patients with non-cancerous disease is different from patients with cancer [4]. More studies comparing TEPs with platelets from patients with non-cancerous inflammatory disease and healthy individuals could give us more insight into the function of platelets in different diseases including cancer. To gain more insight, the collection has been initiated of relevant patient samples from multiple non-cancerous clinical conditions, such as multiple sclerosis, inflammatory bowel disease, chronic obstructive pulmonary disease, cardiovascular disease, pancreatitis, and premalignant lesions (e.g., pancreatic intra-epithelial neoplasms). These cohorts may improve the strength of thromboSeq in the clinical practice by not only comparing healthy from cancer but separating patients with similar clinical symptoms that could either have a benign or malignant disease.

## Future directions

The assessment of the main and alternative approaches for development and implementation of liquid biopsies in the clinical setting requires a strongly interdisciplinary effort with a wide range of scientific and technology competencies to lead a radical breakthrough with transformative impact. Combining TEPs with other biosources may enable next-generation liquid biopsy tests for the detection and specification of cancer. Key will be to get the most out of the individual biosources using the most sensitive techniques, including high-intensity sequencing (perhaps including detection of novel epigenetic and epitranscriptomic features). The newest functional assays may be used to interrogate the different biosources on a functional level, in particular CTCs, TEPs, and EVs [79–81]. Moreover, it may be of interest to combine nucleic acid-based biomarkers with functional assays through state-of-the-art approaches such as targeted NGS for detecting cancer-specific modifications of nucleic acids (RNA/DNA). Detection and usage of cancer-driven alterations of the biological properties of RNA molecules (e.g., nucleotides additions, editing, and modifications) as cancer-specific features may boost a critical new dimension of biomarker discovery. Combining isolation procedures of EVs and ctDNA, TEPs and ctDNA, and CTCs and TEPs have been considered as next-generation biomarker troves [22]. Hence, strong quantitative computational analysis is essential. Through self-learning algorithms, biomarker files can be interrogated to calculate which combination of biomarkers yields the highest sensitivity and specificity. To this end, several computational aspects may need to be taken into consideration, e.g., (i) hierarchically structured and secure data storage and access, (ii) standardized bioinformatics protocols including automated quality controls and primary data analysis, and (iii) innovative machine learning models using different biosource combinations in order to obtain diagnostic synergy and to provide a biological rationale for the functionality of the detected biomarkers.

The thromboSeq technique should be tested in multiple clinical trials before it can be used in a clinical setting. These studies should focus on cancer detection, treatment response prediction, prognostics, or monitoring of disease load. Several studies regarding early detection of cancer have currently implemented platelet RNA analysis in their protocols, e.g., the PLATO-VTE study (ClinicalTrials.gov identifier: NCT02739867) is focused on early detection of cancer in patients with an unprovoked symptomatic pulmonary embolism and/or distal or proximal deep vein thrombosis of the leg. These patients have a 5 to 10% chance of getting the diagnosis of cancer within 1 year after the event. In the VTE-PLATO study, platelet RNA will be analyzed to determine if cancer can be detected earlier, when cure rates are higher.
